# Mr. Jewel on Nitrate of Silver in Leucorrhœa

**Published:** 1830-04-01

**Authors:** 


### XXXVI.

### Mr. Jewel on Nitrate of Silver in Leucorrhœa.

In the Medical and Physical Journal for last October, Mr. Jewel drew the attention of his brethren to the subject of vaginal discharges, and more especially to a remedy not hitherto prescribed in such cases — injections of nitrate of silver. It is Mr. Jewel's opinion that a very common cause of leucorrhœa is a subacute or chronic inflammation of the cervix uteri — and that this phlogosis is not seldom mistaken for more serious affections, as carcinoma uteri. He thinks that the irritable uterus, so ably described by Dr. Gooch, will be remedied by the same plan of treatment which he prescribes for leucorrhœa. The following remarks, he hopes, will assist the young practitioner in his diagnosis of leucorrhœal from more serious diseases of the uterine system.

"This inflammation of the cervix uteri, like scirrhus, or other organic disease of the uterine system, attacks occasionally at the period of life when the catamenia are about to cease ; but I have more frequently found it to exist in married women, from the age of twenty-six or twenty-seven to that of forty, and very recently I have seen several severe cases occurring in young married females, within three months after the birth of the first child. The local symptoms in both diseases are very nearly allied, namely, occasional lancinating pain, more or less acute, through the region of the uterus ; with a constant dull kind of pain about the inferior portion of the sacrum, the hip, or groin; attended also by an irritable bladder, or frequent desire to void the urine, and in some severer cases by tenesmus. The vaginal discharge is of a milky or cream-like colour, and is commonly, but particularly in the more acute cases, mixed with a dark-coloured or grumous secretion. Upon making an examination per vaginam in this disease, the os uteri will not be found opened to the same extent as in carcinoma, nor will its margin present the same cartilaginous hardness to the touch. The pain does not appear to be situated in the edges of the os uteri, as described by Mr. Burns, but in the cervix, as pressure upon this part alone occasions the patient to complain. The uterus will be found projecting lower in the vagina than natural; but this will depend upon the nature of the complaint: the more acute, the farther it will have descended."

Passing over the routine remedies in such cases, Mr. Jewel adverts to the use of nitrate of silver applied directly to the part affected. The mode of application which he has employed is, either to conceal the caustic in a silver tube, precisely on the principle of its application in strictures, or to use a solution of the nitrate, in the proportion of three grains to the ounce of water, gradually increasing the strength. A bit of sponge, firmly and neatly tied to a piece of whalebone, is to be moistened with the solution, and carefully introduced into the vagina up to the os uteri. This mode, he conceives, is preferable to injection, and can be effected by the patient herself. The application should be frequently made. Cases are detailed in illustration, and he concludes his paper by guarding the profession against the idea that he holds up the remedy in the light of a specific. He merely recommends it to their attention as a powerful auxiliary to such other means as the nature of the symptoms may indicate.

In the Westminster Medical Society, held January 23d of the present year, our author again drew the attention of his professional brethren to this subject, and the proceedings of the Society are well reported in the Medical Gazette, of January 30th. In this sitting Mr. Jewel reiterated his opinion respecting the pathology of leucorrhœa, and maintained that it was of an inflammatory, or, at all events, a congestive character. But he queries whether such a condition may not lead to scirrhus or carcinoma uteri. After alluding to constitutional treatment, and especially to iodine, which exerts a powerful influence on the uterine system, Mr. J. reiterated his former experience of the nitrate of silver, and adduced the result of additional observation. This subsequent experience appears to have been confirmative of that which preceded.

From what we have seen of the utility of nitrate of silver in mucous and muco-purulent discharges from other parts of the body—as the urethra of the male, the eye, &c. we have no doubt that it will prove very useful in leucorrhœa, to which disease, we believe, it has not been applied before Mr. Jewel's remarks appeared.

---

